# Quantitative EEG for early differential diagnosis of dementia with Lewy bodies

**DOI:** 10.3389/fpsyg.2023.1150540

**Published:** 2023-04-20

**Authors:** Sandro Iannaccone, Elise Houdayer, Alfio Spina, Gianluca Nocera, Federica Alemanno

**Affiliations:** ^1^Department of Rehabilitation and Functional Recovery, IRCCS San Raffaele Scientific Institute, Milan, Italy; ^2^Department of Neurosurgery and Gamma Knife Radiosurgery, IRCCS San Raffaele Scientific Institute, Milan, Italy

**Keywords:** dementia, quantitative electroencephalography, cognition, EEG, dementia with Lewy bodies

## Abstract

**Introduction:**

Differentiating between the two most common forms of dementia, Alzheimer’s dementia and dementia with Lewy bodies (DLB) remains difficult and requires the use of invasive, expensive, and resource-intensive techniques. We aimed to investigate the sensitivity and specificity of electroencephalography quantified using the statistical pattern recognition method (qEEG-SPR) for identifying dementia and DLB.

**Methods:**

Thirty-two outpatients and 16 controls underwent clinical assessment (by two blinded neurologists), EEG recording, and a 6-month follow-up clinical assessment. EEG data were processed using a qEEG-SPR protocol to derive a Dementia Index (positive or negative) and DLB index (positive or negative) for each participant which was compared against the diagnosis given at clinical assessment. Confusion matrices were used to calculate sensitivity, specificity, and predictive values for identifying dementia and DLB specifically.

**Results:**

Clinical assessment identified 30 cases of dementia, 2 of which were diagnosed clinically with possible DLB, 14 with probable DLB and DLB was excluded in 14 patients. qEEG-SPR confirmed the dementia diagnosis in 26 out of the 32 patients and led to 6.3% of false positives (FP) and 9.4% of false negatives (FN). qEEG-SPR was used to provide a DLB diagnosis among patients who received a positive or inconclusive result of Dementia index and led to 13.6% of FP and 13.6% of FN. Confusion matrices indicated a sensitivity of 80%, a specificity of 89%, a positive predictive value of 92%, a negative predictive value of 72%, and an accuracy of 83% to diagnose dementia. The DLB index showed a sensitivity of 60%, a specificity of 90%, a positive predictive value of 75%, a negative predictive value of 81%, and an accuracy of 75%. Neuropsychological scores did not differ significantly between DLB and non- DLB patients. Head trauma or story of stroke were identified as possible causes of FP results for DLB diagnosis.

**Conclusion:**

qEEG-SPR is a sensitive and specific tool for diagnosing dementia and differentiating DLB from other forms of dementia in the initial state. This non-invasive, low-cost, and environmentally friendly method is a promising diagnostic tool for dementia diagnosis which could be implemented in local care settings.

## 1. Introduction

Alzheimer’s disease (AD) and other forms of dementia are significant causes of disability and dependency among older people, worldwide (Lisko et al., 2021). While no curative therapies are currently available for dementia, there are considerable benefits to the early diagnosis of dementia and early differentiation between dementia subtypes. These benefits include better patient counseling and disease prognostication, appropriate selection of pharmacological and non-pharmacological options for symptomatic management, and early modification of cardiovascular risk factors which adversely affect disease progression. Early disease identification is also considered critical to develop both symptomatic and disease modifying therapies (Rasmussen and Langerman, 2019). The recent approval of aducanumab, a monoclonal antibody targeting amyloid-β fibrils, by the U.S. Food and Drug Administration, has been controversial; however, this potentially disease modifying treatment for AD further emphasizes the need for early and specific diagnosis of AD, as the phase 3 trial evidence for aducanumab suggests that it may exert a clinically significant effect, slowing the progression of cognitive decline in AD, but only in the early phase of the disease (Cummings et al., 2021).

After AD, the most common form of dementia is dementia with Lewy bodies (DLB) (Walker et al., 2015; McKeith et al., 2017; Arvanitakis et al., 2019). Reports suggest that DLB is under-diagnosed in clinical practice (Mok et al., 2004; Toledo et al., 2013) with difficulties in making an early diagnosis and differentiating DLB from AD posing the greatest challenge (Walker et al., 2015). Currently, the diagnosis of DLB is based on the identification of core clinical features: cognitive fluctuations (a particularly difficult clinical feature to elicit accurately), visual hallucinations, parkinsonism, and RBD (McKeith et al., 2017). Supportive clinical features and indicative biomarkers (including Positron Emission Tomography (PET), Single Positron Emission Computed Tomography (SPECT) and Magnetic Resonance Imaging (MRI), electroencephalography (EEG) and polysomnography (PSG)) can provide further indications for the diagnosis of DLB (McKeith et al., 2017). The accurate, early diagnosis of DLB is particularly important in order to ensure the appropriate selection of symptomatic pharmacotherapy as certain medications, namely most antipsychotics, which may be used to manage hallucinations or agitation, can generate potentially severe adverse reactions in approximately half of patients with DLB (Aarsland et al., 2008).

In the DLB diagnosis process, routine clinical assessments including physical examinations, blood tests, and basic neuropsychological tests must generally be supplemented by increasingly specialist assessments such as neuro-immunological analysis of cerebrospinal fluid (CSF), requiring an invasive lumbar puncture, complex neuropsychological and electrophysiological tests requiring specialist expertise and equipment and expensive, resource-intensive neuroimaging assessments (Walker et al., 2015; McKeith et al., 2017). These assessments, though effective, require many heavy resources and are therefore costly, in terms of time, money, and their environmental impact and are often only available in specialist centers. Thus, there is a need to develop and promote the use of robust but inexpensive, sustainable and easy-to-use diagnostic tools which can be implemented in small clinical centers and which can be used to streamline the assessment process, giving an indication of which patients warrant more in depth assessment or indeed a diagnostic tool which could provide a robust diagnosis without the other measures.

Electroencephalography (EEG) is a non-invasive diagnostic method which is relatively simple to implement, is inexpensive and therefore, could be provided in most clinical centers. Quantitative EEG analyses (qEEG), an EEG analysis methodology utilizing different computational algorithms such as fast Fourier transform (FFT) or auto regressive (AR) models, has been shown to be a reliable method for measuring modulations in cerebral activity in dementia, with the ability to differentiate AD from other forms of dementia, such as frontotemporal dementia or DLB (Caso et al., 2012; Engedal et al., 2015). EEG of patients with DLB are characterized by theta and delta activity in the posterior, anterior and temporal regions (van der Zande et al., 2018). Slower background activity has been constantly reported in DLB patients compared to AD with the mean dominant frequency ranging between 6.7–7.5 Hz for DLB and 7.5–8.8 Hz for AD (Law et al., 2020). Moreover, alpha relative power in occipital regions is reduced in AD compared to DLB while delta relative.

Power is higher in DLB than AD (Babiloni et al., 2017, 2018). Increased theta/delta power or activities would be more prominent in the posterior region in DLB patients (Kai et al., 2005; Bonanni et al., 2015; Babiloni et al., 2017). Although the dominant frequency was lower with more pre-alpha activities in the anterior region, the diagnostic accuracy of posterior pre-alpha rhythm was higher in differentiating DLB from AD (Bonanni et al., 2008, 2015, 2016). Studies of connectivity showed that phase lag index within the alpha range was lower in DLB than AD, indicating more severe changes in connectivity in DLB (van Dellen et al., 2015; Dauwan et al., 2018; van der Zande et al., 2018). Analyses of event-related potentials also showed differential abnormalities between DLB and AD patients, with delayed auditory or visual P300 in DLB patients (Bonanni et al., 2010; Kurita et al., 2010). Regarding the early stages of the various forms of dementia, EEG abnormalities have been reported to be more common in DLB, even at the mild cognitive impairment (MCI) stage (van der Zande et al., 2020). Thus, analysis of EEG features might have a good accuracy in differentiating DLB from other forms of dementia (Law et al., 2020). Regarding the association between EEG analyses and DLB clinical symptoms, EEG slowing has been correlated with cognitive fluctuations (Briel et al., 1999; Walker et al., 2000a,b; Stylianou et al., 2018). Hallucinations have been associated with slowing of dominant rhythm and decreased functional connectivity (Dauwan et al., 2018; Aoki et al., 2019). Regarding the relationship between EEG abnormalities and cognitive functions, severity of EEG abnormalities have been shown to correlate with MMSE scores (Law et al., 2020). Moreover, EEG features in DLB patients have been shown to correlate with specific domains of cognitive function, such as fronto-executive and visual abilities. The correlation coefficient values ranged between 0.29 and 0.60 indicating weak to moderate correlations (Law et al., 2020).

Thus, many EEG algorithms have been proposed to investigate the pathophysiology of DLB. Applying qEEG using the statistical pattern recognition (SPR) method (qEEG-SPR), where EEG data are processed and classified based on comparison with normative data from a well-defined group of patients with various dementia disorders and from healthy controls, has been shown to be effective in identifying patients with subjective cognitive decline and MCI that have a high risk of converting to dementia over a 5-year period (Ferreira et al., 2016). Moreover, in the last decade, several studies have applied the qEEG-SPR method in order to identify patterns in AD, DLB or other dementias (Snaedal et al., 2010, 2012; Ommundsen et al., 2011; Engedal et al., 2015; Ferreira et al., 2016). Such methods could distinguish patients with dementia from healthy controls with a sensitivity of 76.9% and a specificity of 73.2%, and, among patients with dementia, to differentiate patients with DLB from other forms of dementia with a sensitivity of 90.9% and a specificity of 91.1% (Ferreira et al., 2016). To this aim, MentisCura have developed and tested in the last decade a qEEG-SPR protocol based on a database of 1,000 EEG recordings of patients with clinically confirmed dementia subtypes and 500 healthy controls (Gudmundsson et al., 2007). This database has been developed to identify various classifiers contrasting different sub-cohorts. These classifiers can then be applied to subsequent EEG recordings, constituting an independent estimate of the properties of the classifiers (Engedal et al., 2015).

In this study, we used retrospective clinical data to assess the use of the MentisCura qEEG-SPR protocol in a real-world sample of patients who had been referred for dementia assessment. We aimed to assess the utility of this protocol in identifying dementia and in distinguishing between DLB and other forms of dementia, using the clinical diagnosis [based on the diagnosis criteria for dementia and DLB diagnosis (American Psychiatric Association and American Psychiatric Association, 2013; McKeith et al., 2017)] obtained at the time of assessment as our diagnostic standard. We also aimed to assess whether the combination of neurological assessment, EEG, and neuropsychological tests could further improve the sensitivity and specificity of the results.

## 2. Materials and methods

### 2.1. Population

Thirty-two patients who visited the outpatient clinic of the Department of Rehabilitation and Functional Recovery of the San Raffaele Hospital (Milan, Italy) with suspected initial state of dementia or cognitive impairment were recruited for this study, as well as 16 healthy controls. To participate to this study, patients had to be aged 50 to 85 y.o. and present symptoms of dementia according to the DSM-5, i.e., substantial impairments in one or more cognitive domains, sufficient to interfere with independence in everyday activities (Hugo and Ganguli, 2014). Oral and written consents were obtained from participants, in accordance with the Code of Ethics of the World Medical Association (Declaration of Helsinki) and the study was approved by the local Ethics committee of the San Raffaele Hospital.

### 2.2. Assessments

Every participant underwent the following visits prior to the inclusion of their data in the study: clinic visit with neurologist, EEG recording visit, and follow-up clinic visit at 6 months. When available, neuropsychological evaluation of patients was gathered for analyses.

#### 2.2.1. Neurological examination and clinical diagnosis

Medical history was obtained from both the patient and a close caregiver, in order to characterize the nature, course, and magnitude of cognitive changes (Arvanitakis et al., 2019). The neurologic examination aimed at identifying objective evidence of neurocognitive issues such as aphasia, apraxia or agnosia, and focal neurologic signs of parkinsonism and included a physical examination to identify systemic vascular disease and systemic signs of rare dementia (Arvanitakis et al., 2019). Based on the neurological examination and all the available data, such as neuropsychological evaluation or MRI/PET data, the neurologist gave a diagnosis of dementia or non-dementia. The majority of MRI or PET imaging was performed in different clinical centers, therefore images were not available for analysis in this study, but clinical reports were used for diagnosis. The diagnosis of probable or possible DLB was based on the diagnostic criteria for DLB (McKeith et al., 2017). A follow-up neurological assessment was performed after 6 months. At both visits, patients were seen by two neurologists, who gave their clinical diagnoses independently. At the time of the study, none of the patients were under benzodiazepines or acetylcholinesterase inhibitors.

#### 2.2.2. EEG recording

EEG recordings were obtained the week following the neurological evaluation. EEGs were recorded from 19 Ag/AgCl electrodes fixed on an elastic cap accordingly to the 10–20 International System, referenced to CPz, with the ground in AFz. The 19 recording electrodes were the following: Fp1, Fp2, F3, F4, C3, C4, P3, P4, O1, O2, F7, F8, T3, T4, T5, T6, Fz, Cz, and Pz. Patients were seated on an armchair, with their arms and legs at rest, and were asked to close their eyes. Five-minute resting-state EEGs were recorded for each patient. Signals were sampled at 1 kHz and coded on 16 bits. Impedances were kept below 5 kΩ. EEG data were acquired using the NicoletOne EEG System from Natus^®^.

#### 2.2.3. Neuropsychological evaluation

Some patients, included in the study, had previously underwent a detailed neuropsychological evaluation. The following tests for different cognitive domains were then analyzed: Mini Mental State Examination (MMSE) (Folstein et al., 1975), Attentive and Raven Matrices (Raven, 2003), Token test [36], Semantic fluency (Novelli et al., 1986), Phonemic fluency (Novelli et al., 1986), naming (Miceli et al., 1994), word picture matching test (Kaplan et al., 1983), Digit span test (Orsini et al., 1987), Digit Span Backward (Wechsler, 1955), Corsi block-tapping test (Corsi, 1972), Rey Complex Figure Test (Carlesimo et al., 1996), Trail making test (Reitan, 1955), Stroop test (Jensen and Rohwer, 1966; Heaton et al., 1993), and Wisconsin Card Sorting test [46].

### 2.3. qEEG data analyses

The analyses methods described below have been employed in previous studies (Snaedal et al., 2010, 2012; Ferreira et al., 2016; Engedal et al., 2020).

The EEG segment used for analysis was selected by a trained technician who chose a segment with minimal presence of artifact and a length of at least 150 s. Prior to feature extraction, the chosen segment was preprocessed by applying an 8th-order Butterworth band-pass filter with the chosen band (0.1–70 Hz) to eliminate potential low- and high-frequency disturbances from the signal. The features extracted from the EEG recording and used in the evaluation of the dementia index (DI) were retrieved according to the recommendations of the Pharmaco-EEG society (Jobert et al., 2013). The society recommends that the signal is segmented into 2-s segments overlapping by 1 s. The signal is then analyzed segment by segment, and the feature values are estimated by evaluating the expected value over all the segments. This can be achieved by various means. For instance, using the average value or an alternative robust measure. Using a robust measure minimizes the impact of outliers and hence reduces the influence of potential signal artifacts. We used the simplest robust estimate, that is, the median of the feature values. The features used were all related to the spectral properties of the recording. Discrete fast Fourier transform was applied to estimate the spectral properties of the signal (Cooley and Tukey, 1965). The analysis relied on the recordings from the 19 electrodes. If the fast Fourier transform components for each of the electrodes, segments, and discrete frequencies considered are denoted by σ_cij_, where c ∈ {1,2,…, 19} indicates the channel, i ∈ {1,…, N} the segment of the N segments considered, and j ∈ {1,…, 90} the discrete frequencies (0.5, 1,…, 45 Hz), the full spectral resolution covariance between channels *c* and *k* is then expressed by xij ck = σ_cij_ × σ^*^_kij_. These covariances constituted the base features used for analysis and evaluation of the classification index values.

The aim of the qEEG-SPR protocol was to sort patients within two classifier indices. The first classifier, the “dementia index” (DI), was constructed to separate healthy individuals from patients presenting with any dementia disorder. This index showed good diagnostic capacity for AD (Snaedal et al., 2012). The second classifier, the “DLB index,” was constructed to detect patients with DLB among the clinical cohort of patients with dementia. To determine the core features relied on, principal components (PCs) were determined based on the Mentis Cura database of EEG recordings. PC analysis was performed on data from dementia subjects in the database. This was done separately for each covariance. PCs were then ranked according to their individual discriminatory properties in separating the subjects in the database. The discriminatory properties were determined according to the area under curve (AUC) of the receiver-operating characteristic curve (ROC). We use the 2 best performing components from each of the covariances to extract the core features used for evaluation of the index. If P_ckαj_ denotes the 2 chosen PCs, α ∈ {1, 2}, for electrode pair (c, k) at frequencies j ∈ {1,…, 90}, the core features considered for analysis then become C_ckα_ = Ei {Σ90 j = 1 xij ck Pckαj}. The PCs can be related to the classical EEG power bands, δ (1–4 Hz), θ (4–8 Hz), α (8–13 Hz), and β (13–30 Hz). Then, PC1 corresponds to the difference between the combined δ and θ power and the β power, while PC2 is a weighted measure of the total power with slightly more emphasis on α and β power. The index value for an individual recording is evaluated from these features by I = Σ_ckα_C_ckα_β_ckα_ + β^A^_1_ A + β^A^_2_ A^2^ + ρ, where A is the age of the subject in years. The classification coefficients β_ckα_, β^A^_i_, and ρ were determined using a combination of genetic algorithms to optimize the number of features used, and SVM (support vector machine), an SPR, was applied in the Mentis Cura database, which contains EEG data from people with various dementia diagnoses and HC. This was done separately for men and women, resulting in separate gender-dependent indices.

Analyses were done with Sigla v.3.3®, by an experimenter blinded for all clinical symptoms, medical history, and diagnosis of patients.

### 2.4. Statistical analyses

Confusion matrices were built to evaluate the performance of EEG algorithm for the diagnosis of dementia and DLB compared to the clinical diagnosis representing current clinical practice (reference category: clinical diagnosis; predictor: EEG results). Neuropsychological assessments were compared between DLB and non-DLB patients using Mann–Whitney test. Measures of concordance between EEG and neuropsychological tests were performed using Cohen’s test. A correlation analysis between MMSE scores and the Dementia Index was performed using Pearson’s correlation test.

Data were considered significant when *p* < 0.05. The commercially available software IBM SPSS Statistics v.23 (IBM Corp.©) was used for all statistical tests.

## 3. Results

### 3.1. Demographic and clinical data

Clinical and EEG data from 32 patients, who were visited in our memory outpatient clinic between September 2019 and January 2021, were utilized for this study. Twenty-four out of 32 patients were male, patients’ mean age was 73.6 ± 7.6 y.o. and their mean education level was 11.7 ± 4.2 y. Sixteen controls were also included in the study (6 female, mean age 70.1 ± 7.6 y.o., mean education level 12.3 ± 5.1 y.).

Patients’ demographic and clinical data are summarized in Table 1.

**Table 1:** Reports demographic and clinical data of all patients, including EEG results for dementia index and DLB index and clinical diagnosis at follow-up.

Patients				EEG results		Clinical diagnosis	
#	Gender	Age (year)	Education (year)	Dementia index	DLB index	DLB clinical criteria	Clinical diagnosis (dementia/DLB)
1	M	70	17	Positive	Inconclusive	Fluctuating cognition: YES, cerebral atrophy; neuropsychological deficits; REM behavior disorders; hallucinations: YES	Dementia: YES. DLB: probable
2	M	71	17	Positive	Positive	Patient with dementia due to hemorrhagic stroke in 2016 + neurosurgical intervention (for evacuation)	Dementia: YES. DLB: NO (hemorrhagic stroke)
3	M	75	13	Inconclusive	Negative	Fluctuating cognition: NO, cerebral atrophy: NO, neuropsychological deficits: YES	Dementia: YES. DLB: NO (Initial Alzheimer)
4	M	76	13	Positive	Positive	Fluctuating cognition: YES, hallucinations: YES; sleep disorders: YES; neuropsychological deficits: YES; parkinsonism: YES	Dementia: YES. DLB: Probable
5	M	80	5	Positive	Positive	Fluctuating cognition: YES, cerebral atrophy: YES; neuropsychological deficits: YES; parkinsonism: YES	Dementia. YES. DLB: Probable
6	M	70	11	Positive	Negative	Fluctuating cognition: NO, hallucinations: NO; sleep disorders: NO; neuropsychological deficits: YES; parkinsonism: NO; PET: positive	Dementia: YES DLB: NO (Alzheimer disease)
7	M	71	13	Positive	Negative	Dementia: YES, Fluctuating cognition: NO, hallucinations: NO; sleep disorders: NO; parkinsonism: NO	Dementia: YES DLB: NO
8	M	69	N/A	Positive	Positive	Dementia: YES, REM disorders: YES; Parkinson: NO	Dementia: YES. DLB: Possible
9	F	68	N/A	Positive	Negative	Dementia: YES, Hallucinations: YES; Parkinson: NO	Dementia: YES. DLB: NO
10	M	71	5	Inconclusive	Inconclusive	Cognitive deficits: yes (mild), Fluctuating cognition: NO, hallucinations: NO; sleep disorders: NO; parkinsonism: NO	Dementia: YES. DLB: NO (Alzheimer)
11	M	69	10	Positive	Inconclusive	Cognitive deficits: yes, Fluctuating cognition: NO, hallucinations: NO; sleep disorders: NO; parkinsonism: NO	Dementia: YES. DLB: NO (frontotemporal dementia)
12	M	56	8	Positive	Positive	Cognitive deficits: YES, Fluctuating cognition: NO, hallucinations: NO; sleep disorders: NO; parkinsonism: YES	Dementia: YES. Probable DLB
13	F	62	N/A	Negative	Non calcolato	Cognitive deficits: YES, Fluctuating cognition: NO hallucinations: NO sleep disorders: NO parkinsonism: NO	Dementia: YES. DLB: NO (frontotemporal dementia)
14	M	80	18	Positive	Positive	Cognitive deficits: YES, Fluctuating cognition: NO; hallucinations: NO; sleep disorders: NO; parkinsonism: NO	Dementia YES. DLB: NO
15	M	76	18	Positive	Negative	Cognitive deficits: YES, Fluctuating cognition: YES; hallucinations: NO; sleep disorders: YES; parkinsonism: NO	Dementia: YES. DLB: probable
16	M	71	N/A	Positive	Inconclusive	Cognitive deficits: YES, Fluctuating cognition: YES; hallucinations: NO; sleep disorders: YES; parkinsonism: NO	Dementia: YES. DLB: probable
17	F	75	13	Inconclusive	Negative	Cognitive deficits: YES, Fluctuating cognition: NO; hallucinations: NO; sleep disorders: YES; parkinsonism: YES	Dementia: YES. DLB: probable
18	F	69	N/A	Positive	Inconclusive	Cognitive deficits: YES, Fluctuating cognition: NO; hallucinations: YES; sleep disorders: NO; parkinsonism: YES	Dementia: YES. DLB: probable
19	M	77	8	Positive	Positive	Cognitive deficits: YES, Fluctuating cognition: YES; hallucinations: YES; sleep disorders: NO; parkinsonism: YES	Dementia: YES. DLB: probable
20	M	77	N/A	Positive	Positive	Cognitive deficits: YES, Fluctuating cognition: NO; hallucinations: NO; sleep disorders: NO; parkinsonism: YES	Dementia: YES. DLB: probable
21	M	75	N/A	Negative	Non processed (negative dementia index)	Cognitive deficits: NO, Fluctuating cognition: NO; hallucinations: NO; sleep disorders: NO; parkinsonism: YES (no tremor)	Dementia: YES. DLB: possible
22	F	80	N/A	Positive	Inconclusive	Cognitive deficits: YES, Fluctuating cognition: NO; hallucinations: YES; sleep disorders: NO; parkinsonism: NO	Dementia: YES. DLB: NO (vascular dementia)
23	M	51	N/A	Negative	Non processed (negative dementia index)	Cognitive deficits: YES, Fluctuating cognition: YES; hallucinations: NO; sleep disorders: NO; parkinsonism: NO	Dementia: YES. DLB: NO
24	M	82	N/A	Positive	Positive	Cognitive deficits: YES, Fluctuating cognition: YES; hallucinations: NO; sleep disorders: YES; parkinsonism: YES	Dementia: YES. DLB: probable
25	M	75	13	Positive	Negative	Cognitive deficits: YES, Fluctuating cognition: NO; hallucinations: NO; sleep disorders: NO; parkinsonism: NO	Dementia: YES. DLB: NO
26	F	73	13	Positive	Negative	Cognitive deficits: YES, Fluctuating cognition: NO; hallucinations: NO; sleep disorders: NO; parkinsonism: NO	Dementia: YES. DLB: NO (Vascular dementia)
27	F	73	13	Positive	Negative	Cognitive deficits: YES, Fluctuating cognition: YES; hallucinations: NO; sleep disorders: YES; parkinsonism: NO	Dementia: YES. DLB: probable
28	M	86	N/A	Positive	Positive	Cognitive deficits: YES, Fluctuating cognition: NO; hallucinations: NO; sleep disorders: YES; parkinsonism: NO	Dementia: YES. DLB: probable
29	M	80	8	Positive	Positive	Cognitive deficits: YES, Fluctuating cognition: NO; hallucinations: NO; sleep disorders: NO; parkinsonism: NO	Dementia: YES. DLB: NO (vascular dementia)
30	F	84	N/A	Positive	Positive	Cognitive deficits: YES, Fluctuating cognition: YES; hallucinations: YES; sleep disorders: YES; parkinsonism: YES	Dementia: YES. DLB: probable
31	M	80	13	Positive	Inconclusive	Cognitive deficits: YES, Fluctuating cognition: NO; hallucinations: NO; sleep disorders: NO; parkinsonism: YES (+ restless legs syndrome)	Dementia: NO. DLB: NO (Parkinson patients with chronic cerebral vasculopathy)
32	M	84	5	Positive	Negative	Cognitive deficits: YES, Fluctuating cognition: NO; hallucinations: NO; sleep disorders: NO; parkinsonism: NO	Dementia: NO. DLB: NO (post ictus)

M: Male, F: Female. Age and education are reported in years.

Clinical diagnoses for each patient, given after neurological examination at the first visit (V1) are listed in Table 1. At V1, 30 out of 32 patients were diagnosed with dementia. All patients were in the initial phase of the disease (symptoms’ onset <1 year). Of those diagnosed with dementia (*n* = 30), 2 patients were diagnosed with possible DLB, 14 with probable DLB and 14 had DLB excluded. No diagnoses were revised at follow-up.

### 3.2. EEG reports

#### 3.2.1. Dementia index

qEEG results were reported as a Dementia Index (positive or negative) and a DLB index (positive or negative). Twenty-six out of 32 patients showed a positive Dementia Index result, 3 patients showed a negative Dementia Index result, and 3 showed an inconclusive result (Table 1). Negative Dementia Index was obtained in 13 out of the 16 controls and inconclusive results were obtained for 3 controls.

When comparing qEEG results and the clinical diagnoses of all participants, the EEG dementia index reported 2 false positive (6.3%) and 3 false negative results (9.4%) in the patients’ group.

**Figure 1:**
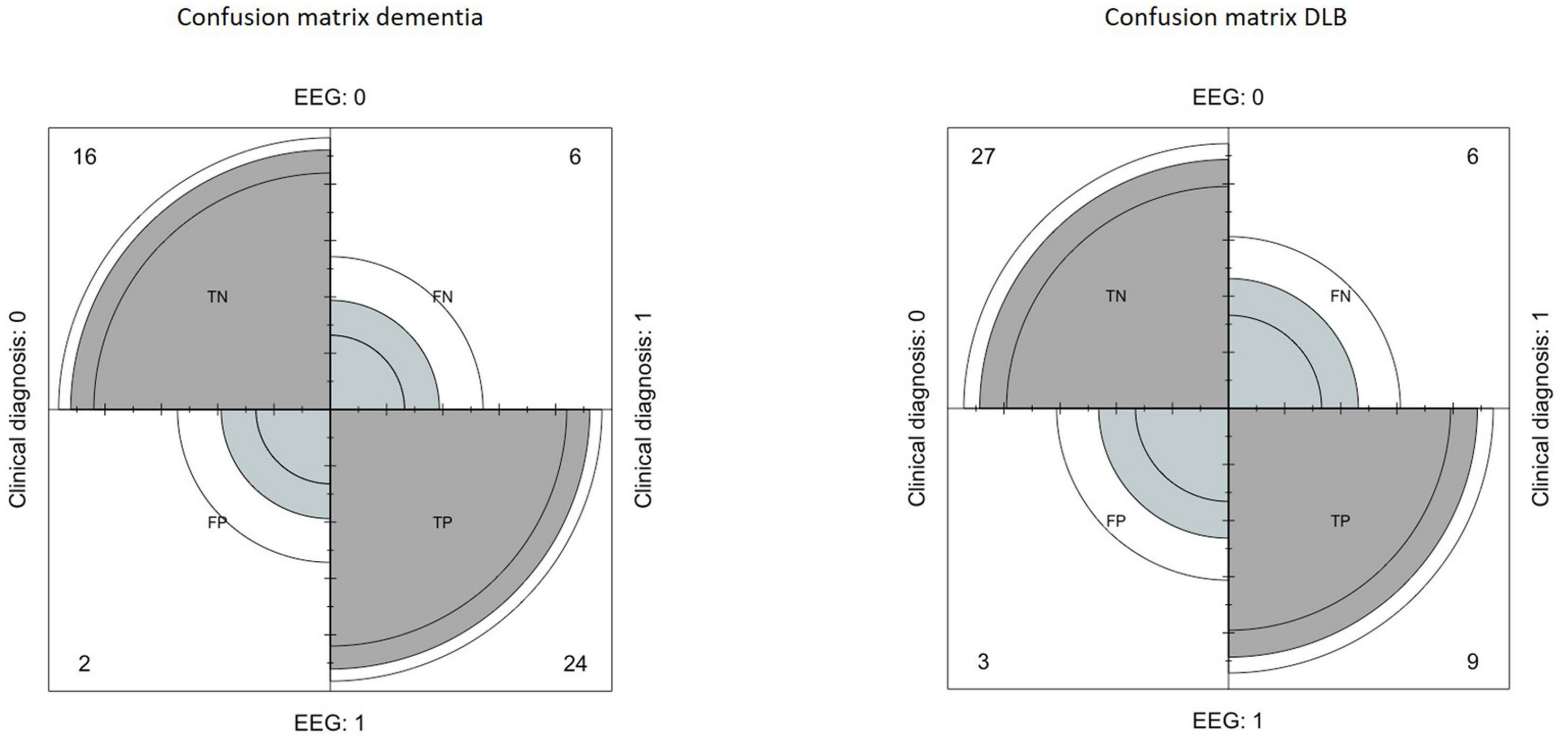
Left: confusion matrix built to evaluate the performance of EEG in the diagnosis of dementia, compared to clinical diagnosis. Right: confusion matrix built to evaluate the performance of EEG in the diagnosis of Lewy Body Dementia, compared to clinical diagnosis.

The confusion matrix indicated a sensitivity of 80%, a specificity of 89%, a positive predictive value of 92%, a negative predictive value of 72% and an accuracy of 83% (Figure 1).

#### 3.2.2. DLB index

Among patients with a positive or inconclusive Dementia Index result (*n* = 29), 12 patients presented with a positive DLB Index result, 10 patients showed a negative DLB Index result, while the DLB index was inconclusive for 7 patients.

Regarding the DLB index, the confusion matrix indicated a sensitivity of 60%, a specificity of 90%, a positive predictive value of 75%, a negative predictive value of 81%, and an accuracy of 75% (Figure 1).

Among the 22 patients who obtained a positive or negative DLB index, 3 patients obtained false positive results (13.6%) and 3 other patients obtained false negative results (13.6%).

#### 3.2.3. Secondary analyses

Two out of the three patients who presented with false positive DLB index had a history of hemorrhagic stroke or head trauma. The third patient suffered from vascular dementia.

We thus removed from the data analyses patients who had a history of stroke or head trauma (*n* = 3). Moreover, since only probable DLB is diagnosed as DLB in clinical settings, we also removed patients with possible DLB diagnoses (*n* = 2).

After removing these patients, when analyzing results of the dementia index, the confusion matrix indicated a sensitivity of 81%, a specificity of 94%, a positive predictive value of 95.5%, a negative predictive value of 76%, and an accuracy of 87.4%.

Regarding the DLB index, the confusion matrix indicated a sensitivity of 657%, a specificity of 96.3%, a positive predictive value of 88.9%, a negative predictive value of 81.2%, and an accuracy of 76.7% of the EEG reports.

### 3.3. Neuropsychological tests

Eighteen out of 32 patients underwent neuropsychological tests. Out of these 18 patients, all were diagnosed with dementia, and 6 out of 18 received a diagnosis of DLB at V1. Neuropsychological scores did not differ significantly between patients with or without DLB (Table 2). Cohen’s coefficient indicated a poor concordance between the EEG reports of dementia (dementia index and DLB index) and neuropsychological test scores (Table 2). The correlation analysis showed a tendency for a negative correlation between the Dementia Index and the MMSE scores (*R* = −0.463, *p* = 0.053).

**Table 2:** Reports median scores and (interquartile range) for neuropsychological tests undergone by 18 out of the 32 patients.

	No DLB	DLB	*p*	Cohen’s significance
Mini mental state examination	22.5 (11)	22 (9)	0.478	poor (−0.1)
Token test	27.75 (8.3)	26.75 (6.9)	0.925	poor (0.11)
Semantic fluency	19 (14)	24 (17)	0.111	poor (0.1)
Phonemic fluency	14 (17)	19.5 (12)	0.205	poor (0.1)
Naming	42 (7)	43 (9)	0.849	poor (−0.1)
Word picture matching test	48 (0)	48 (0)	0.48	poor (0)
Digit span test	5 (2)	5 (1)	0.92	poor (−0.1)
Digit span backward	3 (1)	3 (1)	0.557	poor (0.1)
Corsi block-tapping test	4 (1)	3 (3)	0.13	poor (0.11)
Raven matrices	24.5 (9)	18.5 (14)	0.174	poor (0.03)
Attentive matrices	36 (15)	26 (15)	0.111	poor (−0.11)
Rey complex figure test	30.5 (17)	15.5 (14)	0.061	poor (−0.1)

“*p*” refers to the statistical differences between DLB and non-DLB patients at Mann–Whitney’s testing. Measures of concordance between EEG and neuropsychological tests are reported in the Cohen’s significance column.

## 4. Discussion

This study reports the successful application of machine learning derived indices to a separate and novel clinical dataset. Our data demonstrated that qEEG, using the statistical pattern recognition method, could constitute a sensitive indicator of dementia in a real-world clinical sample and had a high positive predictive value for differentiating DLB from other forms of dementia, even in the initial phase of the disease. This suggests that qEEG has the potential to be a robust method for screening patients for dementia and DLB.

These data confirmed previous evidence showing good sensitivity and specificity for both the dementia and DLB indexes (Engedal et al., 2015, 2020; Ferreira et al., 2016). These diagnoses were confirmed by the clinical examination of patients with clinical diagnoses expressed by two blinded neurologists at V1 and at 6 months follow-up. Our data reported higher sensitivity of the dementia index and lower sensitivity of the DLB index at point value, compared to previous studies (Ferreira et al., 2016). Lower DLB index might have been due to the small number of patients. This method has the advantage to capture multivariate features of the EEG recordings. This leads in general to more robust feature combination allowing for increased test re-test reliability. This particular algorithm of qEEG-SPR utilizes full spectrum analysis of inter-electrode covariances and direct spectral properties at individual electrodes. In this manner, the strategy captures the degrees of freedom related to both classical qEEG features and connectivity/coherence related features through the covariances. The connectivity/coherence related features are functionals of the covariances.

This study demonstrated the robustness and transferability of the qEEG dementia and DLB indices in an outpatient clinic, demonstrating the practical use of such technique for the diagnosis of dementia.

We also observed that the inclusion of patients with a previous history of head trauma, stroke, or neurosurgery might induce false positive results, especially for the DLB index. Indeed, previous evidence has shown that head trauma or chronic stroke can induce long-term changes in the EEG oscillatory activity, such as reduction of the mean alpha frequency or an increase of theta activity (Tebano et al., 1988; Chen et al., 2006; Gosselin et al., 2009; Petrovic et al., 2017; Livint Popa et al., 2020). Similar EEG changes have been evidenced in patients presenting with dementia. In Alzheimer disease, a generalized slowing of the EEG is observed at rest and is expressed by an increased power in the delta and theta frequency bands and a decreased power of the upper alpha and beta bands (Schreiter-Gasser et al., 1993; Huang et al., 2000; Caso et al., 2012). Early EEG slowing may be specific to MCI with Lewy Bodies compared to MCI with AD (Massa et al., 2020; Schumacher et al., 2020). Indeed, in MCI with AD patients, the slowing-down of the qEEG was less severe than in MCI with Lewy Bodies (Massa et al., 2020). These EEG slowing down are especially expressed by the lowering of the alpha/delta ratio. In MCI- DLB, such EEG slowing-down has been mainly observed in the centro-parietal, temporal, and occipital regions (Babiloni et al., 2011; Benz et al., 2014; Massa et al., 2020), although it seems that the slowing observed in the posterior regions might be particularly specific of DLB, compared to AD (Bonanni et al., 2008, 2015, 2016).

Cholinergic deficits, which are more severe and occur earlier in DLB compared to AD, may be the cause of the EEG slowing (Mesulam et al., 2004). EEG frequency is accelerated by cholinergic function and responds to therapy with acetylcholinesterase inhibitors in AD (Fogelson et al., 2003; Babiloni et al., 2013). In DLB, acetylcholinesterase inhibitors may improve global cognitive function, cognitive fluctuations, hallucinations and activities of daily living (Taylor et al., 2020), although only half of patients benefit from this type of treatment (McKeith et al., 2000; Mori et al., 2012; Stinton et al., 2015). These differences in qEEG as well as in EEG connectivity between DLB and AD would explain the strong accuracy of quantitative EEG analyses to differentiate DLB from AD or other dementia (Benz et al., 2014).

Excluding patients with a history of head trauma or stroke, the specificity of the DLB index greatly improved from 90 to 94%. Specificity is of particular importance in the diagnostic process of DLB as it indicates that 94% of the patients with a negative outcome, really do not suffer from DLB. The accuracy of the DLB index also greatly improved from 75 to 87.4%. Such data are similar to previous evidence showing that higher alpha power and lower delta power differentiate AD from DLB with sensitivity and specificity of 65–78% (Babiloni et al., 2017, 2018).

According to clinical criteria for DLB diagnosis, biomarkers are obtained by PET, SPECT, MRI, polysomnographic exams, or EEG. EEG analysis is considered as a supportive biomarker, meaning that EEG data is not considered as indicative as PET or SPECT reports. PET diagnosis of DLB has been shown to have a sensitivity of about 83–92% and a specificity of about 80–87%, similar to previously reported qEEG results (Minoshima et al., 2001; Ishii et al., 2007; Mosconi et al., 2008; Caminiti et al., 2019). Since qEEG has a good accuracy, a high sensitivity and specificity in diagnosing dementia and DLB, considerations could be made to evaluate the possibility to upgrade qEEG analyses from supportive biomarkers to indicative biomarkers. More studies with higher number of patients would be required. Moreover, qEEG analysis represents a diagnostic tool that is non-invasive for the patients, environment-friendly, has a low cost both for the hospital/clinic and patient and can be easily repeated several times a year. Moreover, commercially available software can be used to perform such qEEG-SPR analyses, in order to promote these analyses even in small clinical centers. In these times of pandemic, attention has been brought to patients’ protection and reduction of patients’ displacements to reduce exposure of sensitive populations of patients. Quantitative EEG is a diagnostic tool that can be used even in small clinic centers and could be used as a systematic screening tool for those patients who are suspected of dementia or DLB following neurologic exam and neuropsychological evaluation. Based on the spoke/hub organization of hospitals and clinical centers, patients could undergo neurologic exam, neuropsychological evaluation, and qEEG in spoke centers. Only in the case of positive EEG results, PET/SPECT or MRI could be prescribed in hub centers to confirm the diagnosis.

Our data did not show significant concordance between EEG results and neuropsychological scoring, maybe due to the low number of patients. However, we showed a tendency toward a negative correlation between the EEG Dementia Index and the MMSE scores: the higher the Dementia index, the lower the MMSE. In order to better define such relationship between the Dementia Index and gravity of dementia, such analyses should be reproduced on a larger sample of patients. Evidence has shown that neuropsychological data are highly relevant in dementia and DLB diagnosis (Benz et al., 2014; Zorick et al., 2020; Howard et al., 2021). According to the literature, DLB subjects would have better performance on recall but worse on praxis than patients with AD (Walker et al., 1997). In the early stages, DLB patients present with more visuospatial deficits, compared to AD patients, as shown with the Rosen drawing test (Yoshizawa et al., 2013). Indeed, visuospatial or constructional impairment is present in 74% of patients with early-stage pathologically confirmed DLB compared with 45% of those with AD (Tiraboschi et al., 2006). Moreover, the authors showed that, among clinical variables, history of visual hallucinations was the most specific symptom to DLB (99%), and visuospatial impairment was the most sensitive (74%) (Tiraboschi et al., 2006). MCI patients with AD might have more memory storage impairments, as shown by the Free and Cued Selective Recall Reminding Test (FCSRT), testing for verbal episodic memory (Sarazin et al., 2007). In our study, FCSRT showed a minimal concordance with the EEG results. Such analyses should be replicated on a larger group of patients to further investigate the potential of neuropsychological testing associated with qEEG analyses to improve the sensitivity and specificity of such screening method.

This study presented several limitations, the first of which being the small number of patients. We also showed that certain pathologies or conditions could confound DLB index, such as head trauma or stroke, showing the necessity for exclusion criteria before running qEEG testing. To address these issues, future studies should involve larger cohort of patients, including non-demented patients. Comorbidities should be evaluated to exclude patients with a history of head trauma or stroke. Neuropsychological data should be gathered in all patients to define whether the combination of clinical data, qEEG, and neuropsychological tests could further improve the sensitivity and specificity of differential dementia diagnosis.

## 5. Conclusion

This study confirmed previous findings that qEEG constitutes a highly sensitive and specific tool to perform diagnosis of dementia and differential diagnosis of DLB in the early phase of the disease [12,13,15]. Additional studies, with larger cohorts of patients and control subjects, should be conducted to further confirm the present results to assess whether qEEG algorithms, such as qEEG-SPR, could be upgraded from supportive to indicative biomarker in the process of DLB diagnosis, since its sensibility and specificity are similar to the ones of MRI and PET/SPECT analyses. Quantitative EEG is a non-invasive, low-cost, environment-friendly tool that can be easily installed and used in small clinical centers (spoke). EEG tools should thus be used to streamline the assessment process in dementia diagnosis: EEG exams should be run first and give an indication whether or not more invasive assessments should be undergone to further define the diagnosis. Such invasive assessments are often available only in major clinical centers (hub). Conversely, EEG analyses can be easily implemented in small memory clinics.

## Data availability statement

The original contributions presented in the study are included in the article/supplementary material, further inquiries can be directed to the corresponding author/s.

## Ethics statement

The studies involving human participants were reviewed and approved by Comitato Etico San Raffaele, San Raffaele Scientific Institute. The patients/participants provided their written informed consent to participate in this study.

## Author contributions

SI: participated in study design, data collection, interpretation of data, and paper writing. EH: participated in data collection, data analyses, interpretation of data, and paper writing. AS: participated in data analyses. GN: participated in data analyses. FA: participated in study design, data interpretation, and paper writing. All authors contributed to the article and approved the submitted version.

## Conflict of interest

The authors declare that the research was conducted in the absence of any commercial or financial relationships that could be construed as a potential conflict of interest.

## Publisher’s note

All claims expressed in this article are solely those of the authors and do not necessarily represent those of their affiliated organizations, or those of the publisher, the editors and the reviewers. Any product that may be evaluated in this article, or claim that may be made by its manufacturer, is not guaranteed or endorsed by the publisher.
